# Direct Targeting of the Anterior Nucleus of the Thalamus via 3 T Quantitative Susceptibility Mapping

**DOI:** 10.3389/fnins.2021.685050

**Published:** 2021-07-05

**Authors:** Kaijia Yu, Zhiwei Ren, Tao Yu, Xueyuan Wang, Yongsheng Hu, Song Guo, Jianyu Li, Yongjie Li

**Affiliations:** Beijing Institute of Functional Neurosurgery, Xuanwu Hospital of Capital Medical University, Beijing, China

**Keywords:** anterior nucleus of the thalamus, quantitative susceptibility mapping, deep brain stimulation, epilepsy, anatomy

## Abstract

**Objective:** Deep brain stimulation (DBS) of the anterior nucleus of the thalamus (ANT) is a potentially effective, minimally invasive, and reversible method for treating epilepsy. The goal of this study was to explore whether 3 T quantitative susceptibility mapping (QSM) could delineate the ANT from surrounding structures, which is important for the direct targeting of DBS surgery.

**Methods:** We obtained 3 T QSM, T1-weighted (T1w), and T2-weighted (T2w) images from 11 patients with Parkinson’s disease or dystonia who received subthalamic nucleus (STN) or globus pallidus interna (GPi) DBS surgery in our center. The ANT and its surrounding white matter structures on QSM were compared with available atlases. The contrast-to-noise ratios (CNRs) of ANT relative to the external medullary lamina (eml) were compared across the three imaging modalities. Additionally, the morphology and location of the ANT were depicted in the anterior commissure (AC)-posterior commissure (PC)-based system.

**Results:** ANT can be clearly distinguished from the surrounding white matter laminas and appeared hyperintense on QSM. The CNRs of the ANT-eml on QSM, T1w, and T2w images were 10.20 ± 4.23, 1.71 ± 1.03, and 1.35 ± 0.70, respectively. One-way analysis of variance (ANOVA) indicated significant differences in CNRs among QSM, T1w, and T2w imaging modalities [*F*(2) = 85.28, *p* < 0.0001]. In addition, both the morphology and location of the ANT were highly variable between patients in the AC–PC-based system.

**Conclusion:** The potential utility of QSM for the visualization of ANTs in clinical imaging is promising and may be suitable for targeting the ANT for DBS to treat epilepsy.

## Introduction

Deep brain stimulation (DBS) is a widely utilized and effective treatment for neurologic disorders, including Parkinson’s disease (PD), dystonia, and essential tremor, through the stimulation of deep brain nuclei, such as the subthalamic nucleus (STN) and globus pallidus interna (GPi). DBS is advantageous because it is minimally invasive, reversible, and adjustable. Cooper and Upton first introduced DBS of the anterior nucleus of the thalamus (ANT) and reported the safety and potential efficacy of using DBS to treat refractory epilepsy in several pilot studies (Upton et al., 1985, 1987). In the past decade, ANT-DBS has been used to treat epilepsy in some centers. Results of 56–70% seizure reductions have been reported after the chronic stimulation of the ANT compared with prestimulation baseline during a follow-up of 2–5 years in a controlled, multicenter stimulation of the ANT for epilepsy (SANTE) trial (Fisher et al., 2010). Given the promising prospect of ANT-DBS for the treatment of refractory epilepsy, the accurate identification and targeting of the ANT in brain images prior to DBS surgery is particularly important for neurosurgeons to accurately implant electrodes in the ANT, and the accurate placement of the DBS electrode is closely related to postoperative efficacy (Burchiel et al., 2013; Buentjen et al., 2014; Mottonen et al., 2015; Lehtimaki et al., 2016). Lehtimaki et al. discovered that the implanted lead contacts were located significantly more anterior and superior, both in the anterior commissure (AC)-posterior commissure (PC) and ANT-normalized coordinate systems, in the successful treatment trials performed in 2016 (Lehtimaki et al., 2016). In 2020, they again emphasized the importance of lead placement in ANT-DBS. Contacts that are located more superior inside the ANT were more beneficial than other contacts, especially contacts outside of the ANT. In addition, some patients became responders to ANT-DBS through lead repositioning or changes in the stimulation of contacts. However, other changes, such as changes in stimulation frequency, voltage, pulse width, and stimulation cycle, had minimal effects compared with changes in contact selection (Jarvenpaa et al., 2020). ANT-DBS has been shown to be effective against various epilepsy types, including temporal lobe seizures, frontal lobe seizures, and other types, through long-term follow-up (Krishna et al., 2016). Jarvenpaa et al. (2020) discovered that ANT-DBS resulted in more pronounced effects on focal impaired awareness seizures than on focal aware and focal-to-bilateral tonic-clonic seizures. However, the efficacy of ANT-DBS treatment is often assumed to be limited to those patients with limbic seizures (Fisher et al., 2010).

Currently, 2 methods are utilized for the stereotactic targeting of nuclei, including indirect targeting and direct visualization targeting. In the indirect targeting method, neurosurgeons acquire a coordinate for the ANT location from a brain atlas relative to the anatomical landmark of the midpoint of the AC–PC line. However, this method is lacking in accuracy due to the individual variations in nuclei placement between patients. By contrast, the direct visualization targeting is limited by the poor identification of the ANT on current standard T1-weighted (T1w) and T2-weighted (T2w) magnetic resonance imaging (MRI) sequences, which lack adequate tissue contrast between ANT and its surrounding structures. Some studies have suggested that the boundaries of the ANT can be identified on 3 T MRI T1, 3 T MRI short tau inversion recovery (STIR), and 3 T MRI T1w magnetization prepared gradient echo (MPRAGE) images due to the visualization of the white matter structures surrounding ANTs, such as the external medullary lamina (eml), the internal medullary lamina (iml), and the mammillothalamic tract (mtt) (Buentjen et al., 2014; Mottonen et al., 2015). The absence of an appropriate imaging technique for the direct visualization of the ANT hampers the targeting accuracy of DBS surgery and influences the analysis of efficacy (Jiltsova et al., 2016; Curot et al., 2018). In one study, the responding contacts were overlaid on a template based on an atlas of the ANT, which indicated that a “sweet spot” was located in the inferior and lateral part of ANT, close to the mtt (Krishna et al., 2016). However, individual variations in the anatomy of the ANT were not considered when reporting the results (Buentjen et al., 2014; Mottonen et al., 2015). In addition, Van Gompel et al. emphasized that the ANT is difficult to target reliably and proposed an alternative trajectory via a posterior-inferior parietal approach (Wang et al., 2019).

Quantitative susceptibility mapping (QSM) is a recently developed MRI technique that allows for the better depiction of deep gray matter nuclei *via* the direct measurement of the magnetic susceptibility properties and iron content of intrinsic tissue; this technique differs from the measurement of water relaxation used for conventional MRI (Wang and Liu, 2015). QSM has recently been used in the identification of paramagnetic iron-rich structures, such as the STN and GPi, for DBS surgery, which allowed for the distinction of these structures from the surrounding diamagnetic white matter structures, such as the zona incerta, internal capsule, and medial intermedullary lamina between the GPi and globus pallidum externum (Liu et al., 2013; Yu et al., 2021). Because different subregions of the thalamus have different iron contents, and different degrees of myelinated white matter can be defined in the thalamus, QSM may be useful for delineating the ANT from the enveloping white matter, including the eml, iml, and mtt (Morris et al., 1992; Zhang et al., 2018). In addition, venous structures that contain abundant paramagnetic deoxyhemoglobin can be excellently depicted on QSM, which can help neurosurgeons design trajectories that avoid the veins (Chandran et al., 2016).

This study aimed to explore whether QSM using 3 T units can be used to delineate the ANT from its adjacent white matter structures, providing the direct visualization of the ANT. In addition, information about individual cross-sectional shapes, sizes, locations, and variations in the ANT morphology and positioning of the ANT on the AC–PC-based coordinate system were obtained. Although some studies have reported the superiority of the QSM technique to delineate the STN, GPi, substantia nigra, and thalamus, including the lateral nuclear group, medial group, and posterior group (Liu et al., 2013; Dimov et al., 2018; Corona et al., 2020), few studies have described the use of QSM to delineate the ANT. We propose an approach that uses the QSM technique to provide direct targeting information for use in ANT-DBS surgery to treat epilepsy.

## Materials and Methods

### Subjects

Eleven patients (four men and seven women, mean age: 61.1 ± 5.7 years) with PD (*n* = 6, mean age: 60.0 ± 6.6 years) or dystonia (*n* = 5, mean age: 62.4 ± 4.7 years) who underwent DBS surgery in our center were included as a convenience sample in this study. All patients were deemed to be appropriate candidates for STN-DBS or GPi-DBS surgery after comprehensive preoperative assessments by professional movement disorder neurologists and neurosurgeons. The study was approved by the Hospital Ethics Board, and all patients provided written informed consent before MRI and surgery were performed.

### Data Acquisition

MRI acquisitions, including three-dimensional (3D) T1w, coronal 3D T2w, and a 3D gradient recalled echo (GRE) sequence (3.0 T uMR 770, United Imaging Healthcare, Shanghai, China), were performed on a 3-T MRI scanner several days before the day of the surgery. PD patients with severe tremors at rest were scanned while on medication or were injected with diazepam to reduce the possibility of movement artifacts during MRI examinations. Detailed imaging parameters for T1w, T2w, and GRE sequences, including the repetition time (TR), echo time (TE), bandwidth, field of view (FOV), acquisition matrix, voxel size, and scan time are summarized in Table 1. For QSM map calculation, multiecho GRE data were acquired using the susceptibility-weighted imaging (SWI) + sequence (Ye et al., 2018). B0 field maps were extracted using the multidimensional integration method (Ye and Lyu, 2019), unwrapped from aliased phases using SPUN (Ye et al., 2019b), and background fields were removed using vSHARP (Li et al., 2014). QSM maps were obtained by solving the L1 regularization problem, with an extra term for streaking artifact suppression (Ye et al., 2019a), using the precondition conjugate gradient method. The calculation of B0 and the generation of QSM maps were performed on the postprocessing workstation of the scanner.

**TABLE 1:** Imaging parameters utilized for the T1w, T2w, and GRE sequences.

**Parameter**	**3D T1w**	**3D T2w**	**3D GRE**
Imaging plane	Sagittal	Coronal	Coronal
TR (ms)	7.45	2,000	36.3
TE (ms)	2.5	306	TE_1_/spacing/TE_6_ = 4/5/29
Bandwidth (Hz)	180	650	280
FOV (mm)	256 × 256	256 × 256	200 × 180
Acquisition matrix	256 × 256	384 × 384	400 × 360
Voxel (mm)	1 × 1 × 1	0.67 × 0.67 × 0.67	0.5 × 0.5 × 1
Scan time (min)	4.4	5.4	9

### Image Inspection and Delineation of the ANT on QSM

We first compared the QSM with the Atlas of the Human Brain (4th edition) and the Schaltenbrand and Wahren histologic atlas to qualitatively observe whether the ANT and its surrounding structures, including the white matter laminas and thalamostriate vein (tsv), were visible on QSM (Schaltenbrand and Wharen, 1977; Mai, 2016). For each subject, QSM and T2w were registered to T1w using the rigid-body coregistration method. The degree of geometric distortion on QSM could be roughly evaluated by observing the concordance of the ventricular system, deep brain nuclei, and peripheral cortex. One experienced neurosurgeon (Zhiwei Ren, with 13 years of neurosurgery experience) manually segmented the ANT in each of the three planes (axial, coronal, and sagittal) on QSM with the assistance of two neurosurgeons (Xueyuan Wang, with 12 years of image postprocessing experience, and Tao Yu, with 18 years of experience in epilepsy), who were both familiar with the ANT-DBS surgical procedure used to treat epilepsy. The regions of interest, including ANT and its surrounding eml, were manually depicted as QSM masks, which were also applied to T1w and T2w. The contrast-to-noise ratios (CNRs) of the ANT relative to the eml were calculated to compare tissue contrasts on different MRI scans. The CNR of the ANT was measured using the following equation: CNR = | *S*_*ANT*_ *– S*_*eml*_| /σ, where *S*_*ANT*_ and *S*_*eml*_ represent the mean MR intensities of the ANT and surrounding eml, respectively, and σ represents the measured noise, calculated as the standard deviation of the signal intensity in the medial dorsal thalamus. Because iml and mtt were too thin to select as regions of interest, we did not calculate the CNRs of the ANT relative to the iml or mtt to decrease bias.

In addition, to delineate the morphology and locations of ANT in different patients using QSM, we established a spatial coordinate system using the midpoint of the AC–PC line as the origin. First, QSM was registered with T1w and T2w using the FSL toolbox. The midpoint of the AC–PC line was determined on 3D T1w. Then, the neurosurgeon (Zhiwei Ren) segmented the ANT with manual contours in each of the three planes (axial, coronal, and sagittal) on all slices from QSM, based on AC–PC system. We chose slices where mtt was clearly defined to inspect the ANT, which can assist in the observation of ANT morphology and position on QSM for comparison against the results of conventional images because the mtt is typically used as a marker for targeting the ANT in these images, such as 3 T T1w, 3 T MRI STIR, and 3 T T1w MPRAGE, in other studies. Next, a cross-sectional model of the ANT was calculated according to the manual contours on this slice and was overlaid onto an AC–PC-based coordinate template in the sagittal and coronal orientations. The lengths, widths, heights, and cross-sectional areas of the ANTs were calculated according to these cross-sectional models. Moreover, the anatomical information regarding ANTs from the Schaltenbrand and Wahren histologic atlas and Morel’s atlas were plotted onto the AC–PC-based coordinate system (Schaltenbrand and Wharen, 1977; Morel, 2007). Anatomic variations of ANTs between subjects and different atlases can be compared according to these cross-sectional models in the AC–PC-based coordinate system.

### Statistical Analysis

Statistical analysis was performed using the R studio software (Version 1.1.383). Differences in CNR values (ANT-eml) between the three MRI modalities (QSM, T1w, and T2w) were compared using a one-way analysis of variance (ANOVA). If the one-way ANOVA was significant, *post hoc* tests were performed using independent-sample *t*-tests to evaluate the CNR (ANT-eml) differences between each of the MRI contrasts (T1w vs. QSM, T2w vs. QSM, and T1w vs. T2w). In addition, two-way ANOVAs were performed to examine the significance of the interaction between patient type (PD and dystonia) and imaging modalities (QSM, T1w, and T2w). All results are expressed as the mean ± standard deviation. A *p*-value < 0.05 was considered significant.

## Results

**FIGURE 1:**
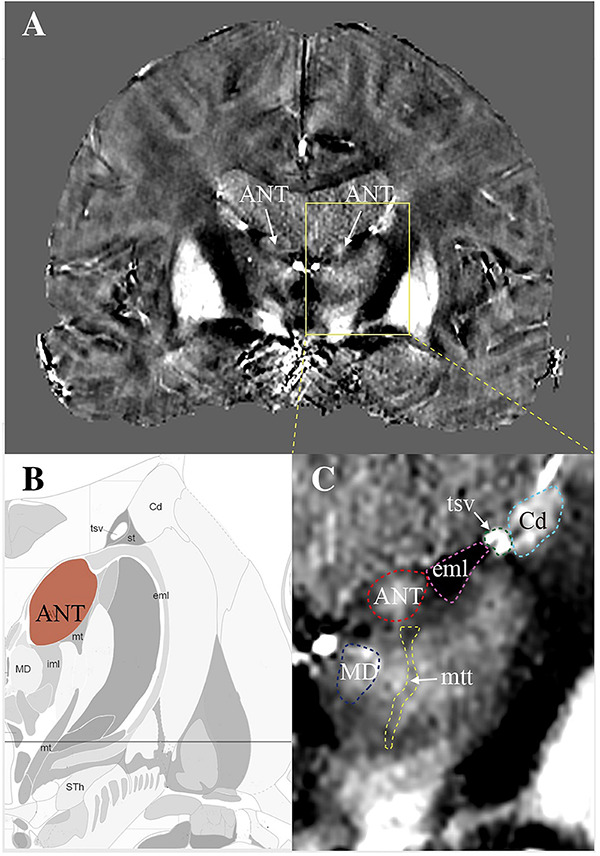
The visualization of ANT on quantitative susceptibility mapping (QSM). **(A)** A coronal view of a slice from QSM to show bilateral ANTs (white arrows) with its surrounding structures. **(B)** A map created according to the image section from the Atlas of the Human Brain in Stereotaxic (MNI) Space (MNI: -13.99). **(C)** Enlarged view of ANT with its surrounding structures. ANT, anterior nucleus of the thalamus; eml, external medullary lamina; mtt, mammillothalamic tract; tsv, thalamostriate vein; Cd, caudate nuclei; MD, mediodorsal nucleus.

**FIGURE 2:**
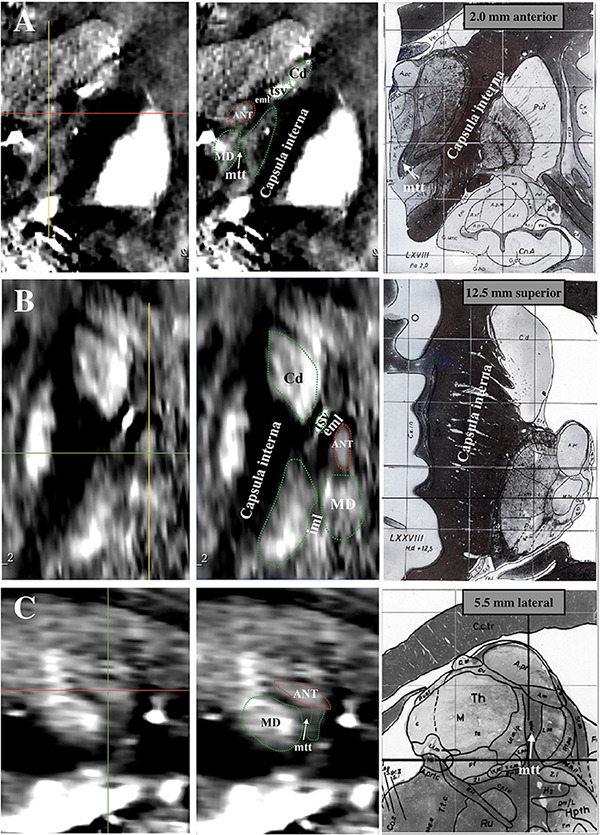
Comparison between Schaltenbrand and Wahren histologic atlas (slices: 5.5 mm lateral; 2.0 mm anterior; 12.5 mm superior) and 3 T QSM. **(A)** Coronal orientation. **(B)** Axial orientation. **(C)** Sagittal orientation. ANT, anterior nucleus of the thalamus; eml, external medullary lamina; iml, internal medullary lamina; mtt, mammillothalamic tract; tsv, thalamostriate vein; Cd, caudate nuclei; MD, mediodorsal nucleus; QSM, quantitative susceptibility mapping.

Figure 1 shows an example map on a coronal imaging section (Figure 1B) from the Atlas of the Human Brain in Stereotaxic (MNI) Space (MNI: -13.99) (Mai, 2016), and the coronal QSM slice (Figures 1A,C) of a representative patient to help recognize the ANT and its surrounding tissue. The paramagnetic ANT was surrounded by relatively diamagnetic white matter tissue, such as the mtt, eml, and iml (Figure 1B). As shown, the coronal QSM provided a clear visualization of the anatomy of the ANT, which presented with an oblique oval appearance, dorsolateral eml, and ventrolateral mtt in the coronal orientation (as indicated by the white arrows). In addition, the hyperintense tsv between the ANT and the caudate nuclei (Cd) was also distinctly identified. Figure 2 presents a detailed comparison of the QSM in coronal, axial, and sagittal orientations with the Schaltenbrand and Wahren histologic atlas. In this study, the ANT appeared hyperintense with regard to the surrounding white matter laminas, and borders were identifiable in all 11 patients in all three orientations. On the coronal images, the superior and lateral borders of the ANT were the most clearly delineated. The mtt that joins the ANT at its inferior border and the eml at the dorsolateral position of the ANT were hypointense and were clearly visible in the coronal orientation. On the axial images, the anterior and lateral borders of the ANT and the eml were clearly identifiable. In some patients, the iml was visible, separating the dorsomedial thalamus and lateral nuclear groups. On the sagittal images, the mtt and the iml inferior to the ANT were occasionally identified due to the level of the image slice. The veins, especially the tsv, which were closely associated with the ANT, appeared with high signal intensity and were distinctly identified in the coronal and axial orientations in all patients. In general, a high degree of consistency was identified for the ANT and its surrounding structures between the QSM and correlating anatomical atlases, which indicated that the ANT and its neighboring structures could be directly targeted on QSM.

**FIGURE 3:**
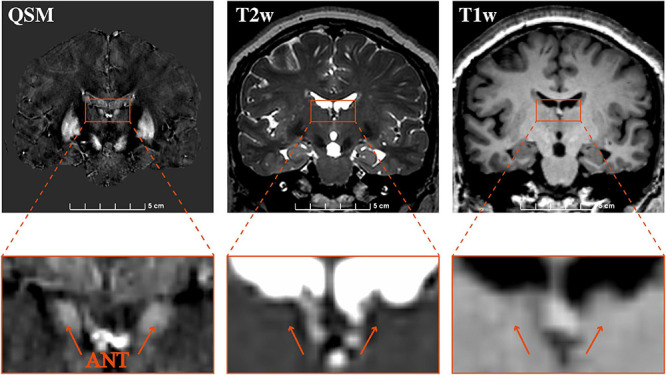
Comparison of the visualization of the ANT on 3 T QSM, T2w, and T1w. Coronal slice views (upper row) and enlarged views of the ANT (lower row) on QSM, T2w, and T1w at one representative section on a representative patient. QSM, quantitative susceptibility mapping; ANT, anterior nucleus of the thalamus.

**FIGURE 4:**
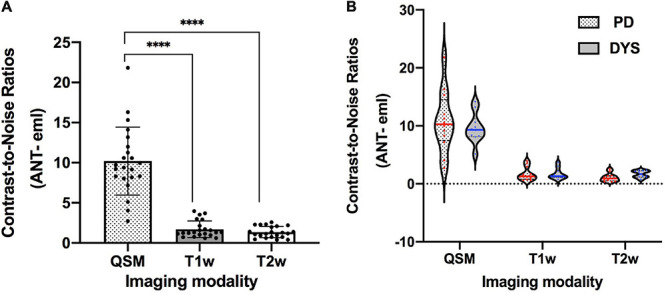
The CNRs of the ANT relative to eml on 3 T QSM, T1w, and T2w. ^*⁣*⁣**^p < 0.0001. **(A)** One-way ANOVA indicated significant differences in CNRs among QSM, T1w, and T2w imaging modalities [*F*(2) = 85.28, *p* < 0.0001]. *Post hoc* tests using independent-samples *t*-tests revealed significant differences in terms of CNRs between QSM and T1w [*t*(21) = 9.156, *p* < 0.0001] and between QSM and T2w [*t*(21) = 9.689, *p* < 0.0001]. **(B)** CNRs (ANT-eml) of different patient types on three imaging modalities were presented using the violin plot. Two-way ANOVA indicated that no interaction effects existed between the patient type and the imaging modality [*F*(2, 60) = 0.614, *p* = 0.544], no significant effect existed for patient type on CNR values [*F*(1, 60) = 0.087, *p* = 0.769], and the imaging modality had a significant effect on CNR values [*F*(2, 60) = 81.10, *p* < 0.0001]. CNR, contrast-to-noise ratio; QSM, quantitative susceptibility mapping; ANT, anterior nucleus of the thalamus; PD, Parkinson’s disease; DYS, dystonia.

The contrasts of the ANT on QSM, T1w, and T2w for one representative patient are compared in Figure 3. On T2w, we can see the blurry borders of the STN, substantia nigra, and globus pallidus. On T1w, the putamen and Cd are nearly localized. However, the location of ANT is difficult to identify on both T1w and T2w. On QSM, the ANT is clearly distinguishable from its surrounding white matter laminas due to differences in susceptibility, as are the distinct borders of the STN, substantia nigra, globus pallidum externum, and GPi. The CNRs of the ANT relative to the eml (ANT-eml) on QSM, T1w, and T2w are 10.20 ± 4.23, 1.71 ± 1.03, and 1.35 ± 0.70, respectively. The one-way ANOVA indicated significant differences in CNRs among the QSM, T1w, and T2w imaging modalities [*F*(2) = 85.28, *p* < 0.0001]. Moreover, *post hoc* tests using independent-samples *t*-tests revealed significant differences in the CNRs between QSM and T1w [*t*(21) = 9.156, *p* < 0.0001] and between QSM and T2w [*t*(21) = 9.689, *p* < 0.0001] (Figure 4A). Two-way ANOVA indicated that no interaction effect existed between the patient type and imaging modality [*F*(2, 60) = 0.614, *p* = 0.544], no significant effect existed for the patient type on CNR values [*F*(1, 60) = 0.087, *p* = 0.769], and the imaging modality had significant effect on CNR values [*F*(2, 60) = 81.10, *p* < 0.0001] (Supplementary Table 1). CNRs (ANT-eml) for different patient types across all three imaging modalities are presented in Figure 4B.

**FIGURE 5:**
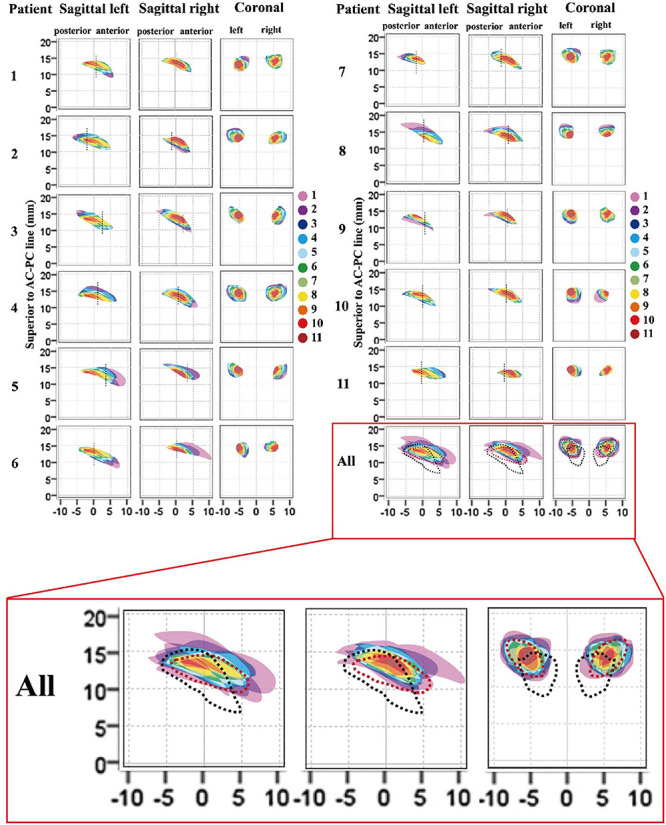
Cross-sectional morphological variations for ANT between patients. Each individual anatomical model (1–11) relative to the whole patient group (all), and the atlas was presented in the AC–PC coordinate system. The degree of overlap (from 1 to 11) for each individual ANT model relative to the whole patient group is represented as different colors. The vertical dashed dark line in the sagittal image represents the location of the coronal ANT model in the *y*-axis. In the last image (all), the degree of overlap in the whole patient group is presented and the ANT models from the Schaltenbrand and Wahren atlas (dark lines) and Morel’s atlas (red lines) are delineated by dashed outlines. The results indicated that the ANTs on 3-T QSM in this group were located more anteriorly, superiorly, and laterally compared with the ANT in the Schaltenbrand and Wahren atlas. However, the locations of the ANT were more consistent with the ANT depicted in Morel’s atlas. ANT, anterior nucleus of the thalamus; AC, anterior commissure; PC, posterior commissure; QSM, quantitative susceptibility mapping.

Cross-sectional models of individual, bilateral ANTs relative to the midpoint of the AC–PC line are presented in Figure 5, based on delineations determined using 3 T QSM in the coronal and axial orientations. Table 2 presents the morphological measurements of the ANT from each cross-sectional model. The mean length of the ANT was approximately 11.25 mm along the anterior-posterior axis of the ANT. The mean height of the ANT was approximately 3.45 mm along the superior-inferior axis of the ANT, and the mean width of the ANT was approximately 4.65 mm perpendicular to the superior-inferior axis of the ANT. The mean cross-sectional area of the ANT on sagittal slices was approximately 26.9 mm^2^, and the cross-sectional area on coronal slices was approximately 16.1 mm^2^. The morphology of the ANT presented a high degree of variation between patients, as indicated in Figure 5. In addition, the location of the ANT also presented a high level of variation using the AC–PC-based coordinate system, as shown in Figure 5. Individual cross-sectional models, which were depicted using different colors corresponding to different degrees of overlap (1–11) relative to the whole patient group in the AC–PC-based coordinate system, are presented in Figure 5. Moreover, ANTs visualized on QSM were typically located anteriorly, superiorly, and laterally relative to the ANT delineated using the conventional Schaltenbrand and Wahren atlas, which is widely used in surgical navigation systems. However, the locations of the ANT were more consistent with the ANT location depicted on Morel’s atlas.

**TABLE 2:** Morphological features of the ANT in study patients.

**Patient**	**AC-PC line (mm)**	**Length (mm)**		**Height (mm)**		**Width (mm)**		**Cross-sectional area**		**Cross-sectional area**	
								**Coronal (mm^2^)**		**Sagittal (mm^2^)**	
		**Left**	**Right**	**Left**	**Right**	**Left**	**Right**	**Left**	**Right**	**Left**	**Right**
1	24.4	10.4	9.6	3.4	3.0	5.4	5.3	17.0	17.1	24.4	20.2
2	23.1	12.0	9.1	3.8	3.8	5.4	5.8	18.1	16.1	34.4	27.9
3	23.0	12.4	12.4	3.8	3.4	3.6	4.0	15.0	16.7	30.8	27.9
4	24.1	12.0	11.2	3.9	3.6	5.1	6.0	23.6	25.1	33.7	31.6
5	23.9	13.4	11.2	4.4	3.4	3.8	4.0	14.3	14.9	39.4	26.3
6	21.0	13.0	13.8	3.4	3.5	4.0	4.3	12.0	10.6	35.8	33.4
7	21.9	8.4	9.8	2.8	3.6	5.6	5.2	18.5	15.7	16.5	23.7
8	23.4	13.4	11.8	3.6	4.2	4.4	4.6	15.8	13.9	34.4	30.6
9	26.4	10.0	9.8	2.8	3.0	5.2	5.0	15.8	14.5	19.7	19.4
10	21.8	14.0	11.0	2.9	3.0	4.8	4.4	21.3	19.2	20.8	21.5
11	23.8	10.4	7.8	3.6	2.6	3.8	3.0	11.4	8.0	24.7	15.0
Mean	23.3	11.8	10.7	3.5	3.4	4.6	4.7	16.6	15.6	28.6	25.2

## Discussion

To the best of our knowledge, although some studies have reported the superiority of the QSM technique for delineating the STN, GPi, substantia nigra, and thalamus, including the lateral nuclear group, medial group, and posterior group (Liu et al., 2013; Dimov et al., 2018; Corona et al., 2020), few studies have explored the use of QSM to delineate the ANT. Our study represents the first attempt to use the recently developed QSM technique to delineate the ANT from the surrounding white matter laminas for use in direct targeting for future ANT-DBS surgery to treat epilepsy. The results demonstrated that the ANT, surrounding tissues, such as the mtt, eml, and iml, and veins could be delineated using 3 T QSM, unlike conventional T1w and T2w imaging techniques that are commonly used for preoperative DBS planning. A higher CNR for the ANT relative to the eml on 3 T QSM was calculated compared with the CNRs calculated for T1w and T2w, suggesting that QSM may be more suitable for the direct targeting of the ANT than T1w and T2w. In addition, variations in the locations of ANTs were studied in the AC–PC coordinate system and compared against the Schaltenbrand and Wahren atlas and Morel’s atlas (Schaltenbrand and Wharen, 1977; Morel, 2007).

Aside from some routine targets used for DBS surgery in clinical treatment, such as the STN for PD and GPi for PD or dystonia, additional targets have been identified with potential effectiveness for the treatment of neurological diseases. The ANT is considered a potential target for epilepsy treatment due to its abundant connectivity and possible functions in the spread of seizure activities. Experimental experience in a pentylenetetrazole rat model has suggested that the ANT plays an important role in seizure spread and generalization (Mirski et al., 1986; Mirski et al., 2003). In a recent, large randomized controlled study of ANT-DBS, a median seizure reduction rate of 41% was achieved during the first year, and a median seizure reduction rate of 69% was achieved at the 5-year long-term follow-up (Salanova et al., 2015). In our previous clinical study, we proposed that desynchronization could be the potential mechanism through which ANT-DBS suppresses seizures, based on data regarding the electrical stimulation of the ANT in epilepsy patients undergoing stereoelectroencephalography examinations (Yu et al., 2018). For DBS surgery, the use of an indirect method to target the ANT using atlas-derived thalamic locations superimposed on the preoperative MRI is not optimal due to variations in the shape, location, volume, and symmetry of ANTs among patients, which can affect the accuracy of electrode implantation and, consequently, decrease epileptic treatment efficacy and increase the risks of adverse side effects and surgical complications. The Schaltenbrand and Wahren atlas is currently the most utilized atlas for stereotactic planning; however, as shown in Figure 5, the ANT in the Schaltenbrand and Wahren atlas (black dotted line) is located more inferiorly, posteriorly, and medially than the ANTs in most of the 11 patients examined in the present study, which indicated that preoperative ANT coordinates acquired using this atlas based on the AC–PC coordinate system should be rectified superiorly, anteriorly, and laterally. In addition, discrepancies existed in the delineation of the ANT in other atlases. Morel’s atlas appears to be more suitable as a reference for the depiction of the ANT than the Schaltenbrand and Wahren atlas, as the location of the ANT in this atlas more closely resembles that of the 11 patients examined in this study based on the AC–PC coordinate system. These result might reflect the iterative approach used to generate Morel’s atlas, in which the mean model was reconstructed from six series of maps derived from different stacks of histologically processed brains, whereas the coronal, sagittal, and axial slices of the Schaltenbrand and Wahren atlas were produced using histological sections from three separate corpses (Schaltenbrand and Wharen, 1977; Morel, 2007).

The direct visualization of the ANT can be helpful for the direct targeting of this nucleus, maximizing the therapeutic benefits and minimizing the potential side effects associated with DBS placement. However, conventional MRI sequences used in the clinic, such as T1w and T2w, at 1.5 or 3.0 T, do not clearly delineate the ANT from surrounding structures due to low image contrast (Buentjen et al., 2014; Jiltsova et al., 2016). With the development of new imaging techniques, studies have reported that 3 T MRI STIR and 3 T MRI T1w MPRAGE can be used to direct the stereotactic targeting of the ANT through the visualization of white matter structures, such as the mtt, eml, and iml, which envelope the ANT (Buentjen et al., 2014). Diffusion tensor imaging sequences have also been postulated as possible methods for tracking the mtt (Grewal et al., 2018). These imaging sequences are not direct methods for visualizing the ANT, but by virtue of visualizing the surrounding structures, they can consequently delineate the ANT borders with relative accuracy. However, we discovered that the superior and medial borders of the ANT were not clearly visible in any of the current state-of-the-art MRI sequences used for DBS targeting of the ANT, including 3 T T1w, 3 T MRI STIR, and 3 T MRI T1w MPRAGE, based on our clinical experience because the white matter typically only wraps around the lateral and inferior borders of the ANT (Buentjen et al., 2014). In addition, the superior border is difficult to distinguish from the choroid plexus in the lateral ventricle of these images (Figure 3). QSM is a novel MRI technique that allows for the better delineation of the deep gray matter nuclei via the direct measurement of intrinsic tissue magnetic susceptibility properties and iron contents. This technique differs from the water relaxation method used to obtain conventional MRI. Recent studies have reported that QSM can be used for the direct targeting of the STN and GPi for purposes of DBS surgery and for the postoperative confirmation of electrode locations relative to nuclei. The QSM technique, when used for the DBS targeting of the STN and GPi, was reliable according to our surgical experience and microelectrode recordings (MER). This technique was superior to the visualization of the STN and GPi using conventional 3 T T1w and T2w and was better for presurgical planning and the verification of lead positioning when combined with the postoperative computed tomography scan (Wei et al., 2019). Due to sufficient iron concentrations in the thalamic nuclei and the different levels of iron deposits detected in different subnuclei, QSM was able to clearly delineate the ANT from its surrounding white matter with adequate tissue contrasts in this study. The ANT appeared hyperintense on QSM due to the paramagnetic susceptibility, and by contrast, the high myelinated white matter, including the mtt, eml, and iml, appeared hypointense relative to the nuclei on QSM due to diamagnetic susceptibility. In addition, the venous structures on QSM appeared more hyperintense than the gray matter due to the presence of deoxyhemoglobin in venous blood. Veins, such as the tsv and the choroid plexus-draining veins, are great limitations when the surgeon is attempting to identify the best trajectory for targeting the ANT. Veins appeared hyperintense on QSM in all patients, which can help surgeons design trajectories that avoid veins without requiring extra vessel contrast agent imaging. In previous studies, two primary transfrontal trajectories were suggested to target the ANT, including the transventricular and the extraventricular trajectories, and both the tsv and the choroid plexus-draining veins must be avoided to prevent intracranial hemorrhage when designing these trajectories (Salanova et al., 2015; Lehtimaki et al., 2019).

The sizes and shapes of the ANTs delineated on 3 T QSM were visually accordant with available atlas information. The nucleus in this study had a length of approximately 11.25 mm, a height of 3.45 mm, and a width of 4.65 mm, which were comparable with the data presented in the atlas. Interindividual variations in the ANT morphology and locations in both sagittal and coronal slices demonstrated the significance of direct targeting based on 3 T QSM data. In addition, hippocampal, amygdala, and thalamic atrophy increased in patients with drug-refractory epilepsy with a longer duration of disease, according to previous volumetric MRI studies (Natsume et al., 2003; Bernasconi et al., 2005). Therefore, the direct targeting of the ANT is a more appropriate method for patients with long-term epilepsy due to the possibility of thalamic atrophy. The direct targeting of the ANT is particularly important in patients who have undergone multiple epilepsy surgeries (especially frontal resection, callosotomies, and temporal resections when the mesiobasal structures are resected). Both the selected target and the selected trajectory can affect the success of electrode implantation in the ANT. Most of the ANTs in the 11 patients assessed by QSM in this study were located more superiorly, anteriorly, and laterally than the ANTs delineated in the Schaltenbrand and Wahren atlas, which indicated that the indirect targeting using a transventricular trajectory might penetrate the primary ANT structure, whereas using a more lateral extraventricular trajectory may only penetrate the inferior segment of the ANT, which could be resolved using a direct visualization targeting approach. In addition, in the previous SANTE trial, DBS lead positions were postoperatively verified by MRI. Contacts located at the center of the ANT were chosen as stimulation contacts, and the lead was replaced if no contact was located in the ANT (Fisher et al., 2010). Some of the stimulation contacts from the SANTE trial may not have been localized in the ANT due to the inconsistency of the ANT localization according to the Schaltenbrand and Wahren atlas compared with individual ANT locations resolved by QSM. Therefore, the clear visualization of the ANT relative to the surrounding structures using 3 T QSM has superiority for accurate preoperative nucleus targeting and postoperative electrode placement confirmation, which should result in better clinical response.

Some limitations must be noted for the current study. First, the present study is based on data obtained from 11 patients with DBS surgery for PD and dystonia, with a mean age of 61.1 ± 5.7 years, which represents an older group than the population of epilepsy patients referred for ANT-DBS based on our clinical experience; therefore, differences between the atlas-based technique and the QSM findings may also be due to age-related differences or differences associated with PD or dystonia presentation. Whether the CNR measurements or ANT morphology observations based on QSM are applicable to the epilepsy population remains unclear. We have occasionally found the ATN to be clearly visible in young, healthy volunteers in figures from other studies using QSM to delineate the STN and GPi, even when using a lower spatial resolution than our study (Liu et al., 2013; Dimov et al., 2018; Cong et al., 2020). Second, image motion artifacts can potentially occur during the scanning of conscious patients with tremors in this study, decreasing imaging quality. The tissue contrast of ANT on QSM might be further improved in patients with epilepsy due to a decrease in motion artifacts. We chose two types of disease (PD and dystonia) as the data set for the present study due to the availability of high-resolution QSM used for DBS targeting in these patients in the clinic. The aim of this study was to preliminarily explore whether QSM could be used to delineate the ANT to apply this technique to patients with epilepsy in the future; thus, we did not restrict the type of disease data. However, our two-way ANOVA indicated no significant main effects of patient type (PD and dystonia) on the CNR values in our sample, which may be attributed to the limitation of the small sample size. The delineation of nuclei on QSM from different types of patients, such as those with PD, dystonia, epilepsy, and other neurological or psychiatric disorders, warrants further study in a large sample size in the future. However, even with the small sample size, the superiority of the QSM technique for delineating the bilateral ANT was still apparent, which indicated that QSM is a promising technique for the direct targeting of the ANT for DBS surgery to treat epilepsy. Third, the T1w had a lower resolution than QSM in this study, which may represent an unfair comparison. The better delineation of ANT in QSM may be due to the higher spatial resolution. Unfortunately, we could not compare the effects of changing the image resolution because the raw GRE data were not retained. However, in Yi Wang’s study using QSM for the depiction of STN, we found that the CNRs of the STN relative to the adjacent tissue were only decreased slightly on QSM when this image was resampled to 2-mm slice thickness from the original 0.5-mm isotropic resolution (Dimov et al., 2018). In addition, the borders of the ANT are clearly visible on 3 T QSM with a spatial resolution of 0.75 × 0.75 × 2 mm or 0.75 × 0.75 × 1.5 mm, based on images from other studies (Liu et al., 2013; Dimov et al., 2018; Cong et al., 2020). Fourth, the accuracy of QSM to delineate ANT was not completely defined, and the measurements were performed on patients in whom the ANT stimulation was not planned. Although QSM shows susceptibility contrast near the ANT based on comparisons with the histologic atlas, whether the border of the ANT on QSM represents the true tissue structural border has not yet been verified. At the present time, it seems that no gold-standard or ground-truth methods exist to verify the accuracy of ATN segmentation except for the use of reference atlases. Although MER has been utilized to verify the borders of the STN, ventral intermediate nucleus, and GPi in DBS surgery, MER is not optimal for the verification of ANT borders for surgery. Few studies have reported on electrophysiological technology in ANT-DBS surgery, and single tract microrecordings have been used to decrease excessive intraventricular bleeding risk due to the abundant veins surrounding the ANT (Mottonen et al., 2016). The only benefit of this recording is the exact delineation of the depth of transventricularly implantation into the ANT for the exact planning of contact locations that may be influenced by a brain shift, according to clinical experience. The consistency of the ANT location described by Morel’s atlas with the highly overlapping sections of the ANT from 11 patients on QSM using the AC–PC coordinate system may support the reliability of QSM to delineate the ANT because this atlas was obtained using an iterative approach by reconstructing a mean model using six series of maps derived from different stacks of histologically processed brains (Morel, 2007). The accuracy of QSM for the direct visualization of the ANT and the beneficial effects produced when using QSM for the direct targeting of ATN-DBS require additional future studies. Finally, QSM requires a high computational load for image post-processing, which can take approximately 10 min to complete. However, the DBS surgery is well separated in time from the MRI used for surgical planning, and 10 min is well within the typical length of an MRI exam. A 9-min QSM scan time in this study may not be practical for tremor patients because motion artifacts and streaking artifacts can easily influence the quality of imaging, resulting in the nuclei borders appearing blurry. One patient with PD had poorly controlled tremors in this sample, and the imaging quality was influenced, resulting in lower CNRs for the bilateral ANT-eml compared with those of other patients (Figure 4). However, the CNRs of the ANT-eml calculated from the QSM from this patient were still higher than the results from T1w and T2w. Decreasing the spatial resolution may be one approach for reducing the scan time. The lowest acceptable imaging resolution for ANT visualization on QSM should be examined in the future.

## Conclusion

In summary, our data suggested that the ANT can be visualized from the surrounding white matter laminas using 3 T QSM on PD and dystonia patients, which is superior to 3 T T1w and T2w. Extensive anatomical variations existed in the ANT from both patients and different atlases, indicating that the direct visualization of the nuclei is likely more suitable for targeting the ANT for DBS surgery compared with indirect targeting methods. Further studies remain necessary to verify the accuracy of QSM to delineate the ANT in patients with epilepsy using large sample size, and the beneficial effects of using QSM to target ANT-DBS surgery for the treatment of epilepsy should be explored.

## Data Availability Statement

The original contributions presented in the study are included in the article/Supplementary Material, further inquiries can be directed to the corresponding authors.

## Ethics Statement

The studies involving human participants were reviewed and approved by the Ethics Board of Xuanwu Hospital. The patients/participants provided their written informed consent to participate in this study. Written informed consent was obtained from the individual(s) for the publication of any potentially identifiable images or data included in this article.

## Author Contributions

KY, ZR, TY, and XW: conception and design. KY, ZR, SG, YH, and JL: acquisition of data. KY, ZR, and XW: analysis and interpretation of data. KY: drafting the article. ZR, JL, and YL: critically revising the manuscript. All authors contributed to the manuscript and approved the submitted version.

## Conflict of Interest

The authors declare that the research was conducted in the absence of any commercial or financial relationships that could be construed as a potential conflict of interest.
