# Supplementary material for: Direct Targeting of the Anterior Nucleus of the Thalamus via 3 T Quantitative Susceptibility Mapping
Source: Front Neurosci. 2021 Jul 5;15:685050. doi: 10.3389/fnins.2021.685050 (PMC8287058; doi:10.3389/fnins.2021.685050)
Supplement: Supplementary file 1 [file Table_1.DOCX]

| **CNR values (mean±SD)** | **QSM** | **T1w** | **T2w** | **Statistics (Two-way ANOVA)** | | |
| --- | --- | --- | --- | --- | --- | --- |
|  |  |  |  | **Patient Type** | **Image Modality** | **Interaction** |
| **PD (n=6)** | 10.71±5.29 | 1.70±1.14 | 1.09±0.70 | *F*(1,60)=0.087  *P*=0.769 | *F*(2,60)= 81.10  *P*<0.0001 | *F*(2,60)=0.614  *P*=0.544 |
| **DYS (n=5)** | 9.58±2.59 | 1.71±0.95 | 1.66±0.62 |  |  |  |

**Table S1. Mean CNR values on QSM, T1w, and T2w images in each type of patients.**

**ANOVA, analysis of variance; PD, Parkinson’s disease; DYS, dystonia.**
